# Human papillomavirus genotypes distribution in 175 invasive cervical cancer cases from Brazil

**DOI:** 10.1186/1471-2407-13-357

**Published:** 2013-07-24

**Authors:** Cristina Mendes de Oliveira, José Humberto Tavares Guerreiro Fregnani, Jesus Paula Carvalho, Adhemar Longatto-Filho, José Eduardo Levi

**Affiliations:** 1 Laboratório de Virologia, Instituto de Medicina Tropical, Universidade de São, Paulo, São Paulo, Brazil; 2 Núcleo de Apoio ao Pesquisador do Hospital de Câncer de Barretos – Fundação Pio XII, Barretos, São Paulo, Brazil; 3 Instituto do Câncer do Estado de São Paulo (ICESP), Faculdade de Medicina, Universidade de São Paulo, Paulo, São Paulo, Brazil; 4 Molecular Oncology Research Center, Hospital de Câncer de Barretos, Barretos, São Paulo, Brazil; 5 Life and Health Sciences Research Institute (ICVS), Health Sciences School, University of Minho, 2ICVS/3B’s - PT Government Associate Laboratory, Braga/Guimarães, Portugal; 6 Laboratório de Investigação Médica (LIM) 14, Faculdade de Medicina, Universidade de São Paulo, Paulo, Brazil; 7 Instituto Nacional de Ciência e Tecnologia do HPV (INCT-HPV), Paulo, São Paulo, Brazil

**Keywords:** Human Papillomavirus (HPV), Invasive cervical cancer (ICC), Brazil

## Abstract

**Background:**

Invasive cervical cancer is the second most common malignant tumor affecting Brazilian women. Knowledge on Human Papillomavirus (HPV) genotypes in invasive cervical cancer cases is crucial to guide the introduction and further evaluate the impact of new preventive strategies based on HPV. We aimed to provide updated comprehensive data about the HPV types’ distribution in patients with invasive cervical cancer.

**Methods:**

Fresh tumor tissue samples of histologically confirmed invasive cervical cancer were collected from 175 women attending two cancer reference hospitals from São Paulo State: ICESP and Hospital de Câncer de Barretos. HPV detection and genotyping were performed by the Linear Array HPV Genotyping Test (Roche Molecular Diagnostics, Pleasanton,USA).

**Results:**

170 out of 172 valid samples (99%) were HPV DNA positive. The most frequent types were HPV16 (77.6%), HPV18 (12.3%), HPV31 (8.8%), HPV33 (7.1%) and HPV35 (5.9%). Most infections (75%) were caused by individual HPV types. Women with adenocarcinoma were not younger than those with squamous cell carcinoma, as well, as women infected with HPV33 were older than those infected by other HPV types. Some differences between results obtained in the two hospitals were observed: higher overall prevalence of HPV16, absence of single infection by HPV31 and HPV45 was verified in HC-Barretos in comparison to ICESP patients.

**Conclusions:**

To our knowledge, this is one of the largest studies made with fresh tumor tissues of invasive cervical cancer cases in Brazil. This study depicted a distinct HPV genotype distribution between two centers that may reflect the local epidemiology of HPV transmission among these populations. Due to the impact of these findings on cervical cancer preventive strategies, extension of this investigation to routine screening populations is warranted.

## Background

Invasive cervical cancer (ICC) is the third most common neoplasia among women worldwide, representing 8.8% of all cancers. Worldwide 607,402 new cases of ICC are predicted to occur by 2015 [1], 493,668 affecting women aged less than 65 years with 320,832 attributable deaths. In Brazil, ICC is the second most common cancer among women and the National Cancer Institute (INCA), agency of the Ministry of Health responsible for cancer prevention and control policies, estimated that 17,540 new cases of ICC would occur in 2012, 2,880 of these affecting the São Paulo State [2]. Brazil’s efforts for ICC prevention are based on a national programme of cervical cancer screening that recommends periodic cervical cytology to women aged 25 to 64 years [2].

Anogenital infection by HPV is associated with benign lesions, like *condylomata acuminata*, and malignant lesions, especially in the uterine cervix [3]. More than 120 HPV types were described based on the isolation of complete genomes [4,5], 40 of these are known to infect the anogenital tract [6] and 12 are classified as carcinogens [7]. Virtually all ICC cases are thought to be preceded by a persistent high-risk HPV genital infection [8,9]. HPV16 and 18 together are responsible for 70% of all ICC cases globally and are the two most frequent HPV types in all geographical regions, while the third most common HPV type varies according to country and population group [10-12]. Several studies investigated HPV genotypes contribution among ICC cases of the Brazilian population. All them reported HPV16 and 18 as the most frequent HPV types [13-16], followed by HPV 31 and 33 [13,14], with exception of the Northeast region, where the third most common HPV type is HPV58, followed by HPV45 [16].

Two prophylactic vaccines against high-risk HPV16 and 18 were licensed, a bivalent (HPV16 and 18) and a quadrivalent one (HPV6, 11, 16 and 18). In Brazil, these two HPV vaccines are available only in private clinics and due to their high cost are not accessible to most of the population. Its inclusion in the national programme of immunization remains under debate [2]. The present study provides an update on HPV genotypes distribution in samples of ICC from patients attending two major reference cancer hospitals in São Paulo State, attempting to identify multiple infections and investigate the association between specific histological types and HPV genotypes. Knowledge of HPV genotypes associated to ICC may contribute to the assessment of the impact of current HPV vaccines on the future incidence of these lesions and assist in the decision making of future prevention strategies.

## Methods

### Study population

Between November 2009 and July 2011 women with a diagnosis of invasive cervical cancer were recruited from two reference cancer hospitals in São Paulo State: Instituto do Câncer do Estado de São Paulo (ICESP) and Hospital de Câncer de Barretos – Fundação Pio XII (HC-Barretos). ICESP is a tertiary public hospital located in São Paulo city, the largest city in South America, with approximately 11 million inhabitants [17]. HC-Barretos is a non-profit hospital sustained by a charity foundation, in the city of Barretos, approximately 450 km distant from São Paulo city, both in the same state. São Paulo city is a well advanced medical center attracting thousands of patients from all over Latin America. It is a cosmopolitan center, home to immigrants from all over the world. In contrast, Barretos is a small city of 115 thousand inhabitants [17] but the HC-Barretos also attracts patients from the whole country, promoting cancer prevention in a vast number of inland cities in the surroundings of Barretos and also in distant remote areas.

At the moment of study enrollment and sample collection all patients were naïve to cancer treatment. One hundred and ninety four patients were initially included in the study and had two biopsies collected: one for the histopathological diagnosis and another for the study. Nineteen patients were further excluded due to absence of confirmation of invasive cervical cancer by histopathology. Reasons for exclusion were: absence of invasive cervical cancer (n = 1), sample in which confirmation of cervical origin was not possible (n = 1), CIN I (n = 1), carcinosarcoma (n = 1), endometrial adenocarcinoma (n = 2), cervicitis (n = 1), carcinoma in s*itu* (n = 11) and sample with normal endocervix and lack of ectocervix (n = 1). Therefore, the study analyzed specimens from 175 patients, between 21 and 86 years of age. The histopathological confirmation was made by the local Pathology laboratories. Stage of the disease was coded according to the International Federation of Gynecology and Obstetrics (FIGO) criteria [18]. All clinical and laboratorial data from the patients were obtained from hospital charts.

The study and the written informed consent signed by all patients or their legal representative were approved by the Research Ethical Committees of Hospital das Clínicas-Faculdade de Medicina da Universidade de São Paulo, Instituto do Câncer do Estado de São Paulo (CAPPesq n° 0451/09) and Hospital de Câncer de Barretos – Fundação Pio XII (protocol n°405/2010).

At ICESP, fresh tumor tissue was collected and immediately introduced into a tube containing Specimen Transport Medium – STM (Qiagen, Gaithersburg, USA) and transported to the Virology Laboratory of the Instituto de Medicina Tropical – Universidade de São Paulo (IMT-USP), where they were stored at −20°C. Tumor tissues collected at HC-Barretos were immediately introduced into a cryopreservation tube and sent to the hospital Tumor Bank, where they were stored in liquid nitrogen, until DNA extraction. After DNA extraction, samples were sent to the Virology Laboratory of IMT-USP where all molecular tests were carried out.

### DNA extraction

Tumor tissue DNA was extracted using QIAamp DNA Mini Kit (Qiagen, Gaithersburg, USA) or NucleoSpin Tissue kit (Macherey-Nagel GmbH&Co, Germany), according to manufacturer’s instructions.

### HPV DNA testing

DNA extracted from the 175 samples were submitted to Linear Array HPV Genotyping Test (LA, Roche Molecular Diagnostics, Pleasanton, USA) for HPV detection and genotyping. This commercial available assay is able to detect 37 HPV types (HPV-6, 11, 16, 18, 26, 31, 33, 35, 39, 40, 42, 45, 51, 52, 53, 54, 55, 56, 58, 59, 61, 62, 64, 66, 67, 68, 69, 70, 71, 72, 73 (MM9), 81, 82 (MM4), 83 (MM7), 84 (MM8), IS39 and CP6108).

### Statistical analyses

Statistical analyses were performed with EpiInfo6 software (http://www.cdc.gov/epiinfo). ANOVA, Yates corrected chi square test or Fisher exact test, as appropriate, assuming two-sided tests and a level of significance of 0.05.

## Results

HPV DNA tests were conducted in tumor samples from 175 women with a histopathological diagnosis of invasive cervical cancer. Three samples from ICESP were considered inadequate due to the lack of β-globin and HPV positive results and were further excluded from the analysis.

Eighty (46.5%) patients were recruited at ICESP and 92 (53.5%) at HC-Barretos. Patient’s age ranged from 21 to 86 with a mean of 51.9 years (95% CI 49.7-54.1). Patients from ICESP (mean 51.1 years, SD = 14.7 and 95% CI 47.8 – 54.4) had similar mean age of the HC-Barretos’ patients (mean 52.6 years, SD = 15.0 and 95% CI 49.5 – 55.7) (p = 0.85). Five patients from ICESP (6.2%) were less than 30 years old. After histological assessment, 144 (83.7%) tumors were classified as squamous cell carcinoma (SCC) and 28 (16.3%) as adenocarcinoma (ADC). The two hospitals had similar frequencies of ADC (ICESP 16.2% versus HC-Barretos 16.3%) (p = 0.99). Women with ADC had a similar age of women with SCC (p = 0.91) (Table 1). Disease staging was defined according to FIGO. Most patients were classified as stage II (44.8%), followed by stage I (24.4%), stage III (18.0%), stage IV (11.0%) and for 1.8% stage was unknown.

**Table 1:** Mean age of the patients by histological diagnosis

	**Mean age (SD)**		
	**Total (n = 172)**	**ICESP (n = 80)**	**HC-Barretos (n = 92)**
ADC (n = 28)	46.6 (14.8)	46.1 (13.2)	47.1 (16.0)
SCC (n = 144)	53.0 (14.7)	52.1 (14.8)	53.7 (14.6)

*ADC,* adenocarcinoma; *SCC,* squamous cell carcinoma; *SD*, standard deviation.

### HPV detection and type-specific distribution

Among the 172 samples that showed a valid result on Linear Array HPV Genotyping Test (LA), 170 (98.8%) were HPV positive. One HPV negative sample was from ICESP and the other one from HC-Barretos. Further investigation with additional amplification methods verified the presence of HPV DNA in those as well (data not shown). Most infections were identified as single infections (75.3%).

**Figure 1:**
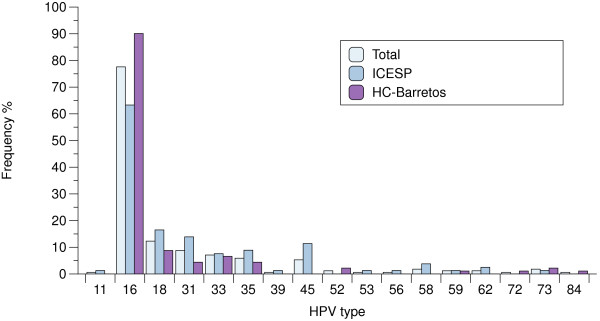
HPV genotypes frequencies in invasive cervical cancer that were positive for HPV DNA.

The frequency of HPV types is illustrated in Figure 1. The five most frequent types were: HPV16 (77.6%), HPV18 (12.3%), HPV31 (8.8%), HPV33 (7.1%) and HPV35 (5.9%) (Table 2). HPV-16 and/or HPV18 were detected in 90.0% of the samples. HPV16 was the most frequent in single infections (77.3%), followed by HPV18 (7.8%), HPV33 (7.0%), HPV31 (3.9%), HPV35 (1.6%) and HPV45, 58 and 59 (0.8%, each). All other HPV types were detected only in multiple infections. One sample from an HIV-infected patient was positive only for low-risk HPVs (HPV11 and HPV62), all others were positive for at least one high-risk HPV type. In both hospitals, HPV16 was the most frequent, followed by HPV18 and HPV31 at ICESP and HPV33 at HC-Barretos. At ICESP, the six most prevalent HPV types were HPV16, HPV18, HPV31, HPV45, HPV35 and HPV33 (Table 2). As single infection, HPV16 was the most prevalent (62.5%) followed by HPV18 (12.5%), HPV31 and 33 (8.9%, each) and HPV35, 45, 58 and 59 (1.8%, each). At HC-Barretos, the most frequent HPV types were HPV16, HPV18, HPV33, HPV31 and HPV35 (Table 2). HPV16 and HPV18 were present in 90.1% and 8.8% of the samples, respectively. HPV16 was the most frequent also as single infection (88.9%), followed by HPV33 (5.6%), HPV18 (4.2%) and HPV35 (1.4%). No HPV31 positive sample was observed as single infection nor any HPV45 positive case was identified at this site. HPV16 was significantly more frequent at HC-Barretos than ICESP, considering only single infections (p < 0.001) and also considering multiple infections (p < 0.001) and HPV45 was significantly more frequent at ICESP than HC-Barretos’ samples (p = 0.02). HPV59 was present in only two adenocarcinoma samples (one as single infection and the other in addition to HPV16).

**Table 2:** HPV genotypes in invasive cervical cancer cases that were positive for HPV DNA

**HPV type**	Total (n = 170)*	**95% CI**	ICESP (n = 79)*	**95% CI**	HC-Barretos (n = 91)*	**95% CI**
**Single infection**	128 (75.3%)	68.1-81.6	56 (70.9%)	59.6-80.6	72 (79.1%)	69.3-86.9
**Multiple infection**	42 (24.7%)	18.4-31.9	23 (29.1%)	19.4-40.4	19 (20.9%)	13.1-30.7
**11**	1 (0.6%)	0.01-3.2	1 (1.3%)	0.03-6.8	--	--
**16**	132 (77.6%)	70.3-83.7	50 (63.3%)	51.7-73.9	82 (90.1%)	82.0-95.4
**18**	21 (12.3%)	7.8-18.3	13 (16.5%)	9.1-26.5	8 (8.8%)	3.9-16.6
**31**	15 (8.8%)	5.0-14.1	11 (13.9%)	7.2-23.5	4 (4.4%)	1.2-10.9
**33**	12 (7.1%)	3.7-12.0	6 (7.6%)	2.8-15.8	6 (6.6%)	2.5-13.8
**35**	10 (5.9%)	2.9-10.5	7 (8.9%)	3.6-17.4	4 (4.4%)	1.2-10.9
**39**	1 (0.6%)	0.01-3.2	1 (1.3%)	0.03-6.8	--	--
**45**	9 (5.3%)	2.4-9.8	9 (11.4%)	5.3-20.5	--	--
**52**	2 (1.2%)	0.1-4.2	--	--	2 (2.2%)	0.3-7.7
**53**	1 (0.6%)	0.01-3.2	1 (1.3%)	0.03-6.8	--	--
**56**	1 (0.6%)	0.01-3.2	1 (1.3%)	0.03-6.8	--	--
**58**	3 (1.8%)	0.4-5.1	3 (3.8%)	0.8-10.7	--	--
**59**	2 (1.2%)	0.1-4.2	1 (1.3%)	0.03-6.8	1 (1.1%)	0.03-6.0
**62**	2 (1.2%)	0.1-4.2	2 (2.5%)	0.3-8.8	--	--
**72**	1 (0.6%)	0.01-3.2	--	--	1 (1.1%)	0.03-6.0
**73**	3 (1.8%)	0.4-5.1	1 (1.3%)	0.03-6.8	2 (2.2%)	0.3-7.7
**84**	1 (0.6%)	0.01-3.2	--	--	1 (1.1%)	0.03-6.0

* Frequency values are above 100% because multiple infections are counted more than once.

**Figure 2:**
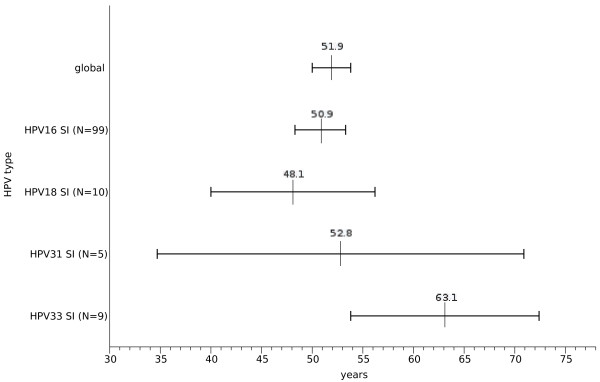
**Mean age (95% CI) at diagnosis of invasive cervical cancer by HPV types.** SI, single infections; HPV, human papillomavirus.

The mean age at diagnosis of invasive cervical cancer of patients infected only by HPV33 are higher than the overall study population and that from patients infected only by HPV16 (Figure 2).

Differences in the relative contributions of HPV types by histological diagnosis were identified. ADC was correlated to HPV59 (p = 0.04) in the study population, but when the analysis was made for each hospital separately no correlation between HPV type and histological diagnosis was observed (Table 3).

**Table 3:** HPV genotypes in invasive cervical cancer cases by histological diagnosis

	**Total**			**ICESP**			**HC-Barretos**		
**HPV Type**	ADC (n = 27)*	SCC (n = 143)*	**p value**	ADC (n = 12)*	SCC (n = 67)*	**p value**	ADC (n = 15)*	SCC (n = 76)*	**p value**
11	--	1 (0.7%)	>0.99	--	1 (1.5%)	>0.99	--	--	
16	21 (65.5%)	111 (76.7%)	0.81	7 (58.3%)	43 (64.2%)	0.95	14 (93.3%)	68 (89.5%)	>0.99
18	6 (20.7%)	15 (7.5%)	0.18	4 (33.3%)	9 (13.4%)	0.21	2 (13.3%)	6 (7.9%)	0.78
31	1 (3.4%)	14 (9.6%)	0.55	1 (8.3%)	10 (14.9%)	0.94	--	4 (5.3%)	0.96
33	--	12 (8.2%)	0.23	--	6 (8.9%)	>0.99	--	6 (7.9%)	0.66
35	1 (3.4%)	9 (6.8%)	>0.99	1 (8.3%)	6 (9.0%)	>0.99	--	4 (5.3%)	0.96
39	--	1 (0.7%)	>0.99	--	1 (1.5%)	>0.99	--	--	
45	2 (17.2%)	7 (3.4%)	0.87	2 (16.7%)	7 (10.4%)	0.82	--	--	
52	--	2 (2.0%)	>0.99	--	--	--	--	2 (2.6%)	>0.99
53	--	1 (0.7%)	>0.99	--	1 (1.5%)	>0.99	--	--	
56	--	1 (1.4%)	>0.99	--	1 (1.5%)	>0.99	--	--	
58	--	3 (2.0%)	>0.99	--	3 (4.5%)	>0.99	--	--	
59	2 (6.9%)	--	0.04	1 (8.3%)	--	0.30	1 (6.7%)	--	0.33
62	--	2 (1.4%)	>0.99	--	2 (3.0%)	>0.99	--	--	
72	--	1 (0.7%)	>0.99	--	--		--	1 (1.3%)	>0.99
73	--	3 (2.0%)	>0.99	--	1 (1.5%)	>0.99	--	2 (2.6%)	>0.99
84	--	1 (0.7%)	>0.99	--	--		--	1 (1.3%)	>0.99

* Frequency values are above 100% because multiple infections are counted more than once.

## Discussion

The present study evaluated geographic differences in HPV genotypes among Brazilian women with invasive cervical cancer recruited at two cancer reference hospitals located at the same region of the country. Some differences between the results obtained in the two hospitals were observed and could be attributable to the fact that these hospitals attend populations with different origins and characteristics, as patterns of sexual behavior (for example the number of sexual partners and age of first intercourse).

Previous studies that evaluated ICC cases from Brazilian women were conducted in all five regions of the country: Eluf-Neto and colleagues (1994) evaluated 199 ICC cases from São Paulo city (Southeast region) [13], Noronha and colleagues (1999) analyzed 155 cases from Belém (Northern region) [14], Rabelo-Santos and colleagues (2003) conducted a study in Goiânia (Central region) and analyzed paraffin-embedded tissues from 56 women [19] and Fernandes and colleagues (2010) carried a study in Natal city (Northeast region) with 88 archival paraffin-embedded tissues from women with ICC [16]. ICC samples (n = 46) from Southern region (Porto Alegre city) of the country were also evaluated in an international study conducted by Bosch and colleagues (1995) [20].

In our study, 98.8% of the samples were HPV positive, corroborating literature data stating that HPV infection is a necessary cause of ICC [8]. Although sensitivity is the most important factor governing HPV detection on ICC due to the usual very low viral load in these cases, other technical caveats may contribute to false negative results, like HPV DNA variability on primers and probe binding sites and partial deletions of HPV genes integrated into cellular genome, among other factors.

The higher HPV prevalence obtained in our study in comparison to others Brazilian studies that obtained rates of 70.3% to 92% [13,14,16,19] and even among studies conducted in different world regions, ranging from 73.1% to 93% [11,20-22], could be explained by the use, in the current study, exclusively of fresh tissue. Several studies used paraffin-embedded specimens that can lead to a lower HPV positivity rate because the fixation process degrades DNA [23,24]. Moreover, Linear Array is one of the most sensitive HPV detection methods available.

In the present study the observed rate of single infections (75.3%) is lower than reported by others [12,16,20,22]. A meta-analysis that included 30,848 cases of ICC worldwide showed that the proportion of HPV multiple infections increased in the last years from 4% to 15% [12], probably reflecting the use of commercial tests more prone to detect multiple infections in recent studies. The high rate of multiple infections reported in the current investigation could be due to the fact that we did not use an automated system in the hybridization step of Linear Array HPV Genotyping Test, what may have allowed some degree of cross-hybridization [25].

Our study confirms the universal contribution of the eight most common HPV types (HPV16, 18, 31, 33, 35, 45, 52 and 58) in ICC samples. Studies conducted in Brazil show that HPV16 is the most common type followed by HPV18 [13,14,16,20], with the exception of the Central region where the second most prevalent type is HPV33 [19]. Another difference in the HPV type prevalence among the different regions of Brazil is verified for HPV58, which is the third most frequent HPV type in the Northeast region [16] but shows a minor contribution in the other country regions. However, comparison of data from different studies shall be taken with caution since there are substantial methodological variations that may have influenced on the overall HPV positivity, as paraffin-embedded versus fresh-frozen tumors, and on the individual rate of HPV types, obviously driven by the inclusion of type-specific detection probes on the post-PCR hybridization steps. The dependence of HPV detection rate on the methodology adopted has been extensively analyzed on the meta-analysis performed by Li et al. [12].

Our data shows that patients from both hospitals had similar mean age, around 50 years old that is in agreement with an international study that evaluated 10,575 ICC cases and reported a mean age of 51.4 years [11]. Brazilian studies conducted in different regions of the country reported a similar mean age, with the exception of Northeast region where patients were younger: Southeast region (52.1 years) [13], Northern region (51.5 years) [14], Central region (49.1 years) [19] and Northeast region (47.3 years) [16].

The majority of samples were histologically classified as SCC cases, while ADC was found in 16.3% of cases, a ratio similar to that described by other studies [11,12], but higher to ADC relative frequencies observed in previous Brazilian studies that observed 3.2% [14], 4.5% [13], 6.8% [15] and 7.1% [19], suggesting that ADC ratio is increasing over time. However, in our study population, women with ADC were not younger than those with SCC, as observed in a study conducted in Peru that reported an increase in the burden of ADC, particularly among young women [26]. This rise in ADCs could be a consequence of well-known limitations of cytology-based screening of ADC precursor lesions since they are frequently located in the endocervical canal, making them less accessible than SCC precursor lesions for cytological detection [27].

We did not observe the correlation of HPV18 and HPV45 with ADC as reported by others [11-13,16]. Our findings associate HPV59, which is related to HPV45 (both belong to the *Papillomaviridae* family alpha-7 species) [4], to ADC. However, HPV16 was also very frequent in ADC cases, especially those from HC-Barretos. Li and colleagues (2011) reported that HPV16 contribution to ADC increased significantly after 2006 [12].

The current series confirms that the detection of exclusive low-risk HPV type in ICC is a rare event (one sample). This case, occurring in an HIV-infected heavily immunosuppressed patient, was described in more details elsewhere and remains unclear if and how a low-risk HPV type may induce ICC [28]. HPV16 and HPV18 were the two most frequent HPV types observed in the present study both in multiple and single infections, in agreement to other Brazilian studies [13,14,16,20], but some differences were observed between the two hospitals: a higher frequency of HPV16, no single infection by HPV31 and no infection at all by HPV45 were observed within HC-Barretos samples. These differences observed between hospitals could be explained by the fact that patients from ICESP included in the present study are from São Paulo city, São Paulo coastal region and also from Northeast region of the country, while HC-Barretos patients are from small countryside cities of the central and southeast region of the country, where there is a lower circulation of people from other regions/countries making the introduction and establishment of new HPV types a rare event. Fourteen different HPV types were observed in the samples from ICESP whereas in the samples from HC-Barretos ten different HPV types were observed. If we compare genotype distribution at ICESP (São Paulo city) in this casuistic compared to that found in samples from 20 years ago [13] it can be observed that HPV 16 and 18 are increasingly frequent, being detected respectively in 53.8% and 8.6% of the older cases and in 63.3% and 16.5% of the current cases. Invasive cervical cancer cases related to HPV33, irrespectively of histological diagnosis, were detected in older women compared to the global study population and to the HPV16 infected subjects.

The women infected by HPV18 and 45 in our study had similar age at ICC diagnosis compared to the global study population, in disagreement with the lower average age of women ascribed to these HPV types reported in other studies [11,22]. This lower average age is attributed to the fact that these HPV types are more likely to integrate into the host genome [29] reflecting a faster tumorigenesis and more aggressive clinical courses. These studies [11,22] also observed a lower average age of ICC diagnosis for women infected by HPV16.

## Conclusions

To our knowledge, this is one of the largest studies employing fresh tumor tissue of invasive cervical cancer cases made in Brazil. The present study confirms the continuing major role of HPV16 and 18 in invasive cervical cancer on recent cases in Brazil, especially at HC-Barretos. Fortunately, available vaccines do include these HPV genotypes, predicting a significant decrease in ICC future incidence over vaccinated women, as only 10.0% of cases would not be target by the current vaccines. Also, ICC screening by existing molecular assays would not require the inclusion of additional HPV genotypes, as they don’t seem to contribute significantly to ICC epidemiology in the Brazilian population.

## Abbreviations

HPV: Human papillomavirus; ICC: Invasive cervical câncer; ADC: Adenocarcinoma; SCC: Squamous cell carcinoma; ICESP: Instituto do Câncer do Estado de São Paulo; HC-Barretos: Hospital de Câncer de Barretos – Fundação Pio XII; INCA: Instituto Nacional de Câncer; FIGO: International Federation of Gynecology and Obstetrics; STM: Specimen transport medium; PCR: Polymerase chain reaction; LA: Linear Array HPV Genotyping Test.

## Competing interests

The authors declare that they have no competing interests.

## Authors’ contributions

CMO was involved in the design of the study, helped in the collection of clinical data, performed the molecular tests and analysis and drafted the manuscript. JHTGF enrolled the study patients at HC-Barretos, collected the biological samples, collected the clinical data and made a critical review of the manuscript. JPC enrolled the study patients at ICESP, collected the biological samples and clinical data and made a critical review of the manuscript. ADF collected the clinical data and made a critical review of the manuscript. JEL conceived the study and was involved in its design, supervised the study, helped to analyze the data and helped draft the manuscript. All authors read and approved the final manuscript.

## Pre-publication history

The pre-publication history for this paper can be accessed here:

http://www.biomedcentral.com/1471-2407/13/357/prepub
